# p67: a cryptic lysosomal hydrolase in *Trypanosoma brucei*?

**DOI:** 10.1017/S003118202000195X

**Published:** 2021-09

**Authors:** Carolina M. Koeller, Terry K. Smith, Andrew M. Gulick, James D. Bangs

**Affiliations:** 1 Department of Microbiology & Immunology, Jacobs School of Medicine and Biomedical Sciences, University at Buffalo (SUNY), Buffalo, NY 14203, USA; 2 Schools of Biology & Chemistry, BSRC, University of St. Andrews, St Andrews, Fife KY16 9ST, UK; 3 Department of Structural Biology, Jacobs School of Medicine and Biomedical Sciences, University at Buffalo (SUNY), Buffalo, NY 14203, USA

**Keywords:** Lysosome, N-terminal nucleophile, p67, phospholipase B-like, trypanosome

## Abstract

p67 is a type I transmembrane glycoprotein of the terminal lysosome of African trypanosomes. Its biosynthesis involves transport of an initial gp100 ER precursor to the lysosome, followed by cleavage to N-terminal (gp32) and C-terminal (gp42) subunits that remain non-covalently associated. p67 knockdown is lethal, but the only overt phenotype is an enlarged lysosome (~250 to >1000 nm). Orthologues have been characterized in *Dictyostelium* and mammals. These have processing pathways similar to p67, and are thought to have phospholipase B-like (PLBL) activity. The mouse PLBD2 crystal structure revealed that the PLBLs represent a subgroup of the larger N-terminal nucleophile (NTN) superfamily, all of which are hydrolases. NTNs activate by internal autocleavage mediated by a nucleophilic residue, i.e. Cys, Ser or Thr, on the upstream peptide bond to form N-terminal *α* (gp32) and C-terminal *β* (gp42) subunits that remain non-covalently associated. The N-terminal residue of the *β* subunit is then catalytic in subsequent hydrolysis reactions. All PLBLs have a conserved Cys/Ser dipeptide at the *α*/*β* junction (Cys241/Ser242 in p67), mutation of which renders p67 non-functional in RNAi rescue assays. p67 orthologues are found in many clades of parasitic protozoa, thus p67 is the founding member of a group of hydrolases that likely play a role broadly in the pathogenesis of parasitic infections.

## Introduction

### The lysosome as a therapeutic target

African trypanosomes (*Trypanosoma brucei* ssp.) are parasitic protozoa that cause human African trypanosomiasis (HAT, aka sleeping sickness), as well as nagana in livestock. These diseases have devastating impact throughout sub-Saharan Africa, wherever the tsetse fly vector is found. A total of >65 million people in 36 countries are at risk of transmission, and although reported human cases have fallen steeply in recent years, it remains a serious veterinary problem. Only a handful of drugs are in use for treating HAT, all of which are either toxic, expensive and/or require a difficult regimen. As vaccination is not possible, and infection is inevitably fatal, there is a critical need for new drug development. Thus, a better understanding of the basic biology of the parasite is essential, particularly of targets amenable to therapeutics. The lysosome is such a target as it impacts the host–pathogen balance in multiple ways. Expression of lysosomal activities is differentially regulated through the life cycle (Caffrey *et al*., 2001), and there are stage specific differences in the biosynthetic trafficking of essential lysosomal components (Alexander *et al*., 2002). The lysosome is the final repository of endocytic cargo acquired from host serum for nutritional purposes (Langreth and Balber, 1975), as well as for potentially lytic immune complexes removed from the cell surface (Balber *et al*., 1979; Barry, 1979). Release of the lysosomal protease cathepsin L (TbCatL) is a factor in the signature event of this fatal human infection, penetration of the central nervous system (Nikolskaia *et al*., 2006). Lysosomal physiology is also critical to the activity of an innate human serum resistance trait, trypanolytic factor (TLF), which limits the mammalian host range of *Trypanosoma* species (Peck *et al*., 2008). Finally, lysosomal hydrolytic activities have considerable potential as chemotherapeutic targets (Selzer *et al*., 1999; Caffrey *et al*., 2011).

### Trypanosome secretory and endocytic architecture

**Fig. 1.**
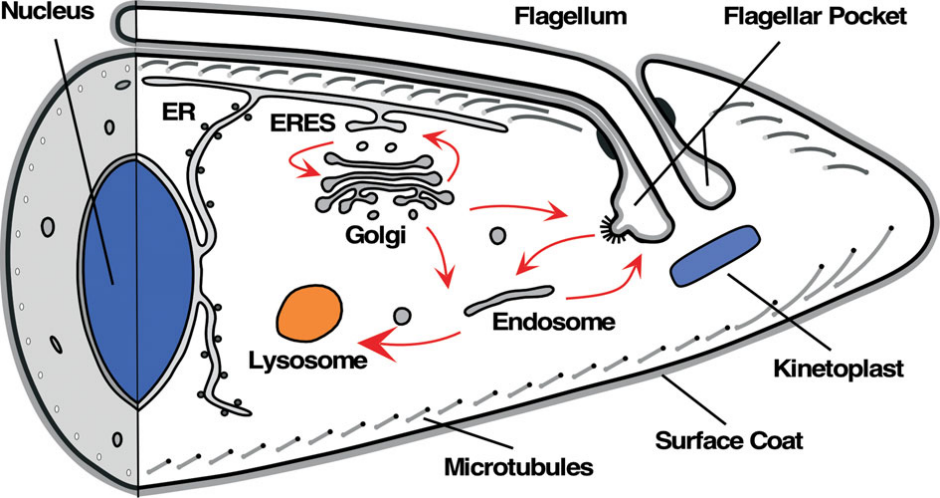
Trypanosome trafficking pathways. Major secretory and endocytic compartments are indicated. Red arrows indicate routes of inter-compartmental trafficking. ‘Endosome’ includes early, recycling and late compartments. The lysosome is a single terminal degradative organelle marked by the soluble thiol protease TbCatL and the transmembrane glycoprotein p67.

Trypanosomes are uniflagellate protozoa with an elongated shape conferred by tightly spaced sub-pellicular microtubules (Fig. 1). Vesicular trafficking of macromolecular cargo, both endocytic and exocytic, is restricted to the flagellar pocket, a small invagination of the plasma membrane at the posterior end of the cell (Landfear and Ignatushchenko, 2001; McConville *et al*., 2002) – all macromolecular cargo going in or out must pass through this restricted domain. The lysosome itself is a single terminal digestive vacuole typically situated just posterior to the centrally located nucleus. In terms of vesicular protein transport, it can be accessed biosynthetically from the Golgi or endocytically from the flagellar pocket *via* endosomal compartments (Engstler *et al*., 2006). There are many markers for the various secretory and lyso/endosomal compartments, in particular early endosome TbRab5A/B (Pal *et al*., 2002), recycling endosome TbRab11 (Morgan *et al*., 2001; Umaer *et al*., 2018) and late endosome TbRab7 (Engstler and Boshart, 2004; Silverman *et al*., 2011). In addition, several components of the ESCRT machinery for sorting from the late endosome to the lysosome have been characterized (Leung *et al*., 2008; Silverman *et al*., 2013; Umaer and Bangs, 2020). The two best characterized lysosomal markers are the major thiol protease TbCatL and the transmembrane glycoprotein p67 (Alexander *et al*., 2002; Peck *et al*., 2008; Tiengwe *et al*., 2018).

## p67: a lysosomal hydrolase?

### p67: history and properties

**Fig. 2.**
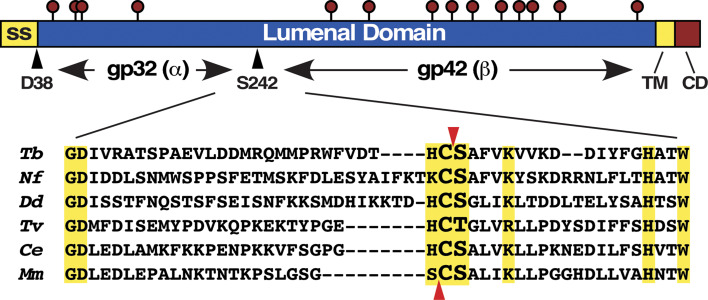
p67 structure and orthologue alignment. (Top) p67 Structure. From N to C termini: signal sequence (ss), lumenal domain, TM and cytoplasmic domain (CD). Lollipops denote *N*-glycosylation sites. The cleavage sites generating the end termini of gp32 (*α*) and gp42 (*β*) subunits are indicated by arrowheads. (Bottom) Alignment of *α*/*β* junction sequences from selected p67 orthologues. Dashes are inserted to allow alignment of regions of high identity (yellow boxes). Known autocleavage sites for p67 (Cys241|Ser242) and mouse PLPD2 (Ser248|Cys249) are indicated by arrowheads (red). Orthologues are: *T. brucei*, Tb927.5.1810; *Naegleria fowleri*, NF0087370; *Dictyostelium discoideum*, DDB_G0271126; *Trichomonas vaginalis*, TVAG_496040; *Caenorhabditis elegans*, NP_499668; *Mus musculus*, NP_076114.

p67 was first identified as a component of total bloodstream form (BSF) trypanosome ricin-binding proteins (Brickman and Balber, 1993). It has a core 67 kDa polypeptide with a type I *trans*-membrane topology (Fig. 2, top): an N-terminal lumenal domain with 14 *N*-glycan sites, a 19 residue *trans*-membrane domain and a 24 residue C-terminal cytoplasmic domain (Kelley *et al*., 1999). Due to topological analogy (there is no sequence homology) to mammalian LAMPs (lysosomal-associated membrane proteins) p67 was originally annotated as ‘LAMP-like’. Biosynthesis, processing and transport of p67 has been studied extensively (Brickman and Balber, 1994; Kelley *et al*., 1995; Alexander *et al*., 2002; Tazeh and Bangs, 2007). It is synthesized in the endoplasmic reticulum as a 100 kDa (gp100) glycoform and during transit of the Golgi in BSF trypanosomes some of these glycans are modified with *N*-acetyllactosamine generating an ~150 kDa (gp150) intermediate glycoform (Fig. 2, top). At least some of these modifications are of the unusually large poly-*N*-acetyllactosamine variety found only in BSF trypanosomes (Nolan *et al*., 1999; Atrih *et al*., 2005), accounting for the large increase in size. Such processing does not occur in procyclic form (PCF) trypanosomes. Upon arrival in the lysosome, p67 is converted to two quasi-stable fragments (N-terminal gp32 and C-terminal gp42) that remain non-covalently associated (Kelley *et al*., 1999). The N-termini of gp32 and gp42 were determined by Edman degradation to be Asp38 and Ser242, respectively, with Asp38 being at the signal sequence cleavage site. The C-termini are not known. Generation of gp32 and gp42 is blocked by FMK024, a selective thiol protease inhibitor (Alexander *et al*., 2002), and by RNAi silencing of TbCatL (unpublished data), indicating TbCatL-mediated cleavage of p67 in the lysosome.

Post-Golgi trafficking of p67 to the lysosome in PCF trypanosomes is dependent on canonical di-leucine repeats in the cytoplasmic domain, on the AP1 clathrin adaptor complex, and presumably on clathrin itself, although this was not tested directly (Tazeh *et al*., 2009). Thus, normal biosynthetic trafficking apparently follows a typical clathrin-mediated pathway to the lysosome. This is likely to be the case in BSF trypanosomes as well, but as p67 still arrives at the lysosome with normal kinetics when the cytoplasmic domain is deleted, it is impossible to state this with certainty. However, this so-called ‘default’ trafficking apparently follows a distinct route since knock downs of both the late endosomal ESCRT component TbVps4 and early endosomal TbRab11 dramatically reduce trafficking of native p67, but have no effect on the deletion mutant lacking the C-terminal domain (Silverman *et al*., 2013; Umaer *et al*., 2018).

RNAi studies showed that p67 is essential in BSF parasites, but the only overt phenotype was gross enlargement of the lysosome to an extended vacuole easily seen by light microscopy (diameter ~250 to >1000 nm), and containing much internal membranous material as seen by transmission electron microscopy (Peck *et al*., 2008). Interestingly, fluorometric assays indicated that the normal lysosomal pH (pH 4.8) was unaffected. However, p67 knockdown, though ultimately lethal, gave temporal protection against both TLF and suramin (Peck *et al*., 2008; Alsford *et al*., 2012). TLF is a toxic subset of human high density lipoproteins, and suramin is a longstanding drug used to treat stage I infections (Hajduk *et al*., 1989; Fairlamb, 2003). Both are taken up by receptor-mediated endocytosis and activated in acidic endolysosomal compartments. Although unsatisfying, it appeared that p67 has an ill-defined role in maintaining lysosomal integrity, and that its loss somehow leads to dysregulation, engorgement and/or swelling. As discussed below, evidence now points to p67 being a member of the N-terminal nucleophile (NTN) superfamily, with possible phospholipase (or other hydrolase) activity.

### N-terminal nucleophiles

The NTN superfamily of hydrolases was first proposed based on the common *αββα* core structure of three distinct enzymes – all amidases (Brannigan *et al*., 1995). Since then membership has grown to include antibiotic acylases, proteasomal subunits, peptidases, quorum quenchers, siderophore maturases, acid ceramidases, snake venom phospholipases, and the phospholipase B-like (PLBL) (see below) (Oinonen and Rouvinen, 2000; Pei and Grishin, 2003; Lakomek *et al*., 2009; Bokove *et al*., 2010; Drake and Gulick, 2011; Coronado *et al*., 2018). All of these enzymes autoactivate by *cis*-cleavage, in which an internal side chain (Cys, Ser or Thr) makes a nucleophilic attack on the upstream carbonyl group leading to peptide bond hydrolysis creating N-terminal *α* and C-terminal *β* subunits. The subunits remain non-covalently associated, and the upstream linker region is typically trimmed, by a second round of autocatalysis or by another protease as proposed for cephalosporin acylase (Kim *et al*., 2002) and murine PLBD2 (Lakomek *et al*., 2009), respectively, although this order of activation has never been shown formally. Linker removal opens up the catalytic site, and the exposed N-terminal amino acid of the *β* subunit then serves as both the general base and nucleophile for hydrolysis reactions. Most known NTNs have aminohydrolase (amidase) activities, but phospholipase B (acylase) activity has been claimed (see below).

### Lysosomal PLBLs

At the time of our initial studies of p67 there were no obvious orthologues in the non-redundant database (Kelley *et al*., 1999; Alexander *et al*., 2002), but shortly thereafter (unbeknownst to us) a family of PLBL enzymes was identified in *Dictyostelium*, mice and humans (Morgan *et al*., 2004; Kollman *et al*., 2005; Xu *et al*., 2009), and all of these reports noted orthology to p67. At some later date (circa 2012) this orthology was also noted in the TriTryp database. The first PLBL characterized was in *Dictyostelium* (DDB_G0276767) (Morgan *et al*., 2004). It was purified from source using a phospholipase assay, and cloned by microsequencing of tryptic peptides followed by reverse transcription-polymerase chain reaction of the corresponding gene. Both native and recombinant protein hydrolysed glycerophospholipids at the *sn*-1 and *sn*-2 positions, consistent with phospholipase B activity. Localization and post-translational processing were not determined. The mouse orthologue, PLBD2 (NP_076114, *nee* lysosomal 66.3 kDa protein) was identified by proteomics of mannose-6-phosphate selected lysosomal proteins, and lysosomal localization was confirmed by immunofluorescence (Kollman *et al*., 2005; Deuschl *et al*., 2006). PLBD2 is a soluble lysosomal protein with five *N*-glycans. Much like p67, it is processed from a 75 kDa precursor to non-covalently associated 28 and 40 kDa N-terminal (*α*) and C-terminal (*β*) subunits, analogous to gp32 and gp42 of p67, respectively. The N-terminus of the 40 kDa *β* subunit is C249, which is equivalent to C241 of p67 (Fig. 2, bottom). Subsequently, the orthologue from human neutrophils was also shown to have PLB activity (Xu *et al*., 2009).

Homology searches identify two paralogues (PLBD1 and PLBD2) in mice, humans and other mammals, and many more orthologues throughout the Eukaryota, often with multiple paralogues in a given species, e.g. eight in *Trichomonas* and five in *Entamoeba* (Table 1). Interestingly, there are no orthologues in the *Crithidia*/*Leishmania* trypanosomatid lineages, indicating secondary loss between *Bodo saltans* (up to 12 paralogues) and *T. brucei* (two paralogues). Most orthologues are soluble proteins; all have conserved Cys/Ser dipeptides at the *α*/*β* junction mapped for p67 and PLBD2 (Fig. 2, bottom, arrows), the only two species for which this has been determined (Alexander *et al*., 2002; Lakomek *et al*., 2009). One notable difference between these two orthologues is that the N-terminus of the gp42/*β* subunit in p67 is Ser242, but in PLBD2 it is the equivalent of the upstream Cys241 (Fig. 2, bottom, arrows). If correct this would make the Ser residue the nucleophile for autocleavage and subsequent catalysis in p67, while the Cys249 residue would play these roles in PLBD2. This could have implications for the catalytic specificities of the two enzymes.

**Table 1.** p67 (PLBL) orthologues

Organism^a^	Accession no.	*E* value^b^	No. of genes	Topology
***Trypanosoma brucei***	**Tb927.5.1810**	**0**	**2**	**Type I TM**
*Bodo saltans*	BSAL_46190	3 × 10^−155^	12	Type I TM
*Naegleria fowleri*	NF0087370	3 × 10^−65^	4	Soluble
*Dictyostelium discoideum*	DDB_G0271126	3 × 10^−58^	7	Soluble
*Entamoeba histolytica*	EHI_069380	2 × 10^−44^	5	Soluble
*Giardia lamblia*	GL50803_93548	2 × 10^−52^	3	Soluble
*Trichomonas vaginalis*	TVAG_496040	3 × 10^−43^	8	Type I TM
*Caenorhabditis elegans*	NP_499668	2 × 10^−63^	3	Soluble
*Mus musculus*	NP_080082.1	3 × 10^−59^	2	Soluble

a There are no orthologues in leishmanial kinetoplastids suggesting secondary loss between bodonids and trypanosomatids. The *Trypanosoma cruzi* orthologue is essentially identical to *Trypanosoma brucei* (*E* value 0).

b BlastP *vs* Tb927.5.1810, best hit only.

The first indication that all these orthologous proteins are NTNs came from the crystal structure of mouse PLBD2, which fits neatly into this superfamily (Lakomek *et al*., 2009). Based primarily upon the enzymatic data from the *Dictyostelium* and human orthologues these proteins have been annotated as PLBs. However, this designation has been challenged by the fact that most NTN enzymes are amidases, not esterases, and because the PLBD2 active site may be too small to accommodate typical phospholipids (Repo *et al*., 2013). In this view, the apparent phospholipase activity is ascribed to either contamination or off-target catalysis. Currently this issue remains an open question.

### Is p67 a PLBL?

**Fig. 3.**
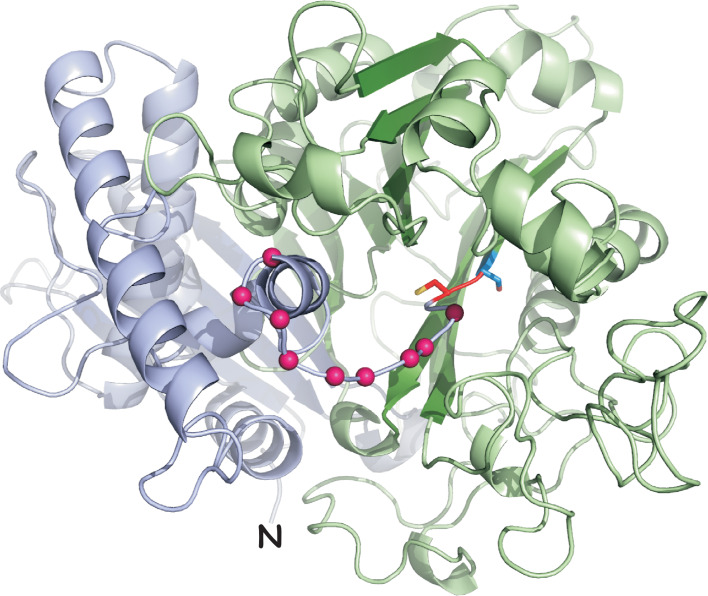
Homology model of the p67 lumenal domain. The p67 sequence was modelled onto the murine PLBD2 structure (PDB: 3FGW) (Lakomek *et al*., 2009) using MODELLER (Webb and Sali, 2017). The gp32(*α*) N-terminus is indicated (N), the C-terminus of gp42(*β*) is hidden. Violet, gp32(*α*) subunit; red, C241; blue, S242; green, gp42(*β*) sub-unit. A plausible model for the unordered linker region is indicated with magenta beads. All *N*-glycan sites model to water accessible surfaces (not shown).

**Fig. 4.**
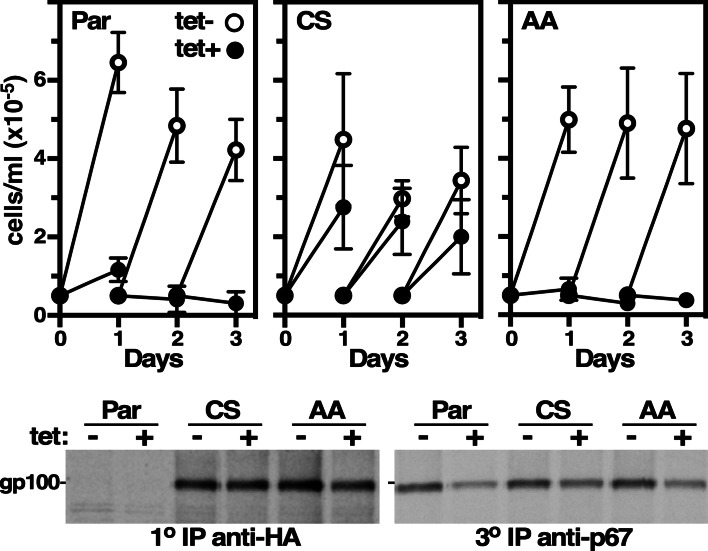
Rescue of p67 RNAi. (Top) The parental p67 RNAi cell line, and derivative cell lines constitutively expressing either HA-tagged RNAi-resistant wild type p67^CS^ (CS) or mutant p67^AA^ (AA), were cultured ±tetracycline to induce dsRNA synthesis. Cells were counted and diluted to starting density every 24 h. Data are mean ± s.d. (*n* = 3) for a representative clone (1 of 3). (Bottom) p67 RNAi was induced for 24 h. Cells were [^35^S]Met/Cys labelled (15 min) and sequential immune capture was performed. Lysates were first immunoprecipitated with anti-HA (1^0^) for recombinant p67, then reprecipitated with anti-HA (2^0^, not shown) to clear residual p67^CS^ and p67^AA^, and finally reprecipitated with anti-p67 (3^0^) to collect remaining native p67. A representative experiment (*n* = 3) is presented.

Is there more evidence that p67 is an NTN of the PLBL subgroup? First, the p67 sequence, despite only 28% identity, models tightly onto the PLBD2 crystal structure (Fig. 3). Importantly all the *N*-glycosylation sites map to the water accessible surface of the model. Second, we have generated recoded RNAi-resistant (RNAi^R^) wild type and double mutant p67 genes, each with a C-terminal HA-tag for discrimination from the native protein. The mutations are Cys241Ala/Ser242Ala spanning the known N-terminus of gp42. The wild type and mutant RNAi^R^ genes are designated p67^CS^ and p67^AA^, respectively. These have been constitutively expressed in an inducible p67 RNAi cell line targeting the endogenous gene product. Upon induction of RNAi the parental cell line ceases growth over a 24 h period (Fig. 4, top). Growth is rescued by the wild type p67^CS^ gene, but not by mutant p67^AA^. RNAi^R^ protein is present in both p67^CS^ and p67^AA^ cells, and silencing reduces endogenous p67 in all cell lines (Fig. 4, bottom, ~60%). Importantly, both RNAi^R^ reporters localize to the lysosome by IFA (not shown). These results establish that one or both of the residues at the gp32/gp42 junction is essential for p67 function. Collectively, these data provide compelling supportive evidence that p67 is an NTN of the PLBL subgroup: (i) lysosomal localization; (ii) conserved autocatalytic residues; (iii) similar biosynthetic processing to *α* (gp32) and *β* (gp42) subunits; (iv) good structural modelling and (v) essentiality of C241/S242 at the gp32/gp42 junction.

## Future directions and conclusions

p67 is an essential lysosomal membrane protein in African trypanosomes. For many years, it was assumed to play an ill-defined role in maintenance of lysosomal physiology – until recently when homology to the PLBL class of lysosomal hydrolases has been recognized. These enzymes are part of the NTN superfamily, but are poorly studied in any system. There is evidence that they do in fact have PLB activity (Morgan *et al*., 2004; Xu *et al*., 2009), but this has also been challenged based upon the fact that all well characterized NTNs are amidases (Repo *et al*., 2013). In either case it is likely that p67 actually is a critical lysosomal hydrolase in trypanosomes, which in turn provides a possible explanation for the main phenotype of p67 knockdown – grossly swollen lysosomes. If p67 is a lipase its loss could result in failure to catabolize glycerophospholipids taken up in host serum lipoproteins leading to membrane engorgement. Alternatively, if it is an amidase, e.g. a peptidase, failure to breakdown lysosomal substrates could lead to solute accumulation with commensurate osmotic swelling. These phenotypes are reminiscent of mammalian lysosomal storage diseases as a consequence of specific enzyme deficiencies (Sun, 2018).

The reassignment of p67 as a lysosomal hydrolase raises many questions – foremost being what is its enzymatic activity? The answer to this question will come from lipidomic and metabolomic analyses of RNAi silenced cells, and these studies are currently underway. Elevated levels of any specific metabolite or lipid will point to the likely substrate(s), and this in turn will provide opportunities for enzyme assay development, and perhaps even small molecule inhibitor screens. A broader question is what are the enzymatic activities of orthologues from other parasitic protozoa, and from mammals? The two *T. brucei* paralogues are essentially identical, except for the transmembrane domain (TM), and hence are likely to have the same activity. On the contrary, the two mammalian orthologues are only 32% identical and are likely to have different substrate specificities. That said, there may be considerable redundancy. PLBD1^−/−^ and PLBD2^−/−^ knockout mice are viable, and at least in the PLBD1 knockout there are no overt phenotypes (http://www.mmrrc.org/catalog/sds.php?mmrrc_id=49098, http://www.mousephenotype.org/data/genes/MGI:1914107, http://www.jax.org/strain/034167). This could explain why no lysosomal storage diseases have ever been associated with mutations in these genes. The situation in other parasitic protozoa is likely to be even more complex given the greater number of paralogous genes in the individual species. The *T. brucei* p67 RNAi cell line may provide a novel approach to defining these specificities. Xeno-complementation (rescue) of the RNAi phenotype by orthologous genes from other parasitic protozoa would indicate overlapping enzymatic activity with p67. Failure to complement would suggest a different activity/substrate specificity, and in this case constitutive expression in trypanosomes, in conjunction with ‘Omics’, could provide insights into enzymatic activity. Most interesting would be xeno-complementation with the human PLBD genes, which might provide insights into the possibility of specific therapeutic targeting of p67 in trypanosomes. Finally, all of these efforts would be augmented by structural studies that push beyond modelling on the known PLBD2 structure, and these efforts are also underway.

Whatever comes of these future experiments the outlook for research on this understudied group of enzymes is bright. For too long p67 has remained a protein without a function, but it is likely soon to take its rightful place as the founding member of a novel class of important lysosomal hydrolases.
